# Rheumatism and Gout

**Published:** 1903-02-07

**Authors:** 


### Rheumatism and Gout.

(*Concluded from page* 305.)

**Spondylitis deformans**, or the spinal variety of arthritis deformans, is one of the oldest known diseases, since instances have been found in Egyptian mummies. Boggs[10] describes a case in a man, aged 45, who had suffered for 33 years, at first with pains in the limbs and then with joint attacks. He had lost all movement in the spine, and nearly all in the hips and jaw. There was complete anchylosis of the thorax, and most of these patients suffer from severe pain due to pressure on the nerve trunks. The hands, feet, and knees in this patient were quite free while the entire spine was immovable.

**Treatment.**—Aspirin is highly praised by Fried. Wielsch[11] after employment in hundreds of cases for its pleasant taste, the absence of gastric irritation, of head troubles and cardiac depression, and its satisfactory action on the joint affections. Even in gonococcal arthritis it relieves the pains to some extent. Gassner also recommends the drug for its efficiency and the absence of secondary effects. Its property of relieving pain is very valuable in numerous disorders, and he attributes its activity to its elimination being slower than that of sodium salicylate. This is probably the case with salol, salophen and similar compounds, and it would be interestlng to have their physiological action accurately compared. Menzer[12] has treated obstinate cases of rheumatism with a streptococcal serum obtained from cocci grown in the tonsils of rheumatic patients. When injected into the body it supplies bacteriolytic matter, though it cannot neutralise the toxins. It does not lessen the fever, but quickens convalescence and prevents relapses.

**Gout** is said to be less frequent in America than in England. The difference, however, is not great according to the Johns Hopkins and St. Bartholomew's statistics which show about one-third more cases in London. Besides the smaller consumption of strong beer in the States, the American is of a mixed race and by no means purely English in origin. Lead workers there furnish a fourth of the cases. Futcher[13] remarks that the output of uric acid increases during an attack and drops below normal afterwards. In one chronic case at Johns Hopkins no uric acid was excreted during the intervals. Seventy-five per cent. of their patients showed organic kidney disease. Cabot found that gout is very rare in Boston, and in the negro it is hardly ever seen. A. P. Luff[14] found that in several gouty persons the alkalinity of the blood was actually higher than in healthy ones by one-third. Since this is due to sodium carbonate which quickens the crystallisation of sodium biurate, it is easy to see the importance of the fact. Potassium salts and especially the citrates are valuable in treatment, both for their action in delaying this change and in other ways. Lithium salts cannot cause this delay and they depress the heart's action unfavourably. It is all important to cure hepatic inactivity, and on this account, where no gouty deposits exist, sodium salts, such as sulphate of soda, may be given, together with blue pill and euonymin. A brisk purge is often more successful than a hypnotic for sleeplessness. Guaiacum resin may be given to prevent the return of the attacks, in doses of 5 grains, to be increased to 10 grains three times a day. No hard and fast rule can be laid down as to diet. Baths and massage are of use in the more chronic cases if the kidneys act well. In neuritis superheated air followed by the constant current gives good results. For gouty eczema we must forbid all alcohol, give blue pill and salines, and employ a careful dietary, avoiding everything which causes dyspepsia, as well as acid fruits, even if ripe, and rhubarb. Colchicum is not required here, but bismuth and an alkaline mixture before meals. Trunek's serum has been recommended for several forms of arthritis. Levy gives it by the mouth or as an enema before breakfast.[15] The formula is as follows :—

Sodium chloride, gm. 10.
Sodium sulphate, 1.
Calcium sulphate }
Magnesium phosph. } aa ·75.
Sodium carbonate, ·40.
Sodium phosph., ·30.

Mix. Divide into 13 powders.

[10] Johns Hopkins Hosp. Bullet., May. [11] Clin. Excerpts, June–Sept. [12] Med. Rec., Sept. 6. [13] Med. News, June 28. [14] Med. News, May 28. [15] Med. Rec., Sept. 13.

